# Enhancing human islet xenotransplant survival and function in diabetic immunocompetent mice through LRH-1/NR5A2 pharmacological activation

**DOI:** 10.3389/fimmu.2024.1470881

**Published:** 2024-09-27

**Authors:** N. Cobo-Vuilleumier, P. I. Lorenzo, E. Martin Vazquez, L. López Noriega, R. Nano, L. Piemonti, F. Martín, B. R. Gauthier

**Affiliations:** ^1^ Andalusian Center of Molecular Biology and Regenerative Medicine (CABIMER), Junta de Andalucía-University of Pablo de Olavide-University of Seville-Consejo Superior de Investigaciones Científicas (CSIC), Seville, Spain; ^2^ Diabetes Research Institute, Istituto di Ricovero e Cura a Carattere Scientifico (IRCCS) Ospedale San Raffaele, Milan, Italy; ^3^ Centro de Investigación Biomédica en Red de Diabetes y Enfermedades Metabólicas Asociadas (CIBERDEM), Madrid, Spain

**Keywords:** xenotranplantation, human islet, nuclear receptor, LRH-1, NR5A2, pharmacological treatment, type 1 diabetes

## Abstract

The intricate etiology of type 1 diabetes mellitus (T1D), characterized by harmful interactions between the immune system and insulin-producing beta cells, has hindered the development of effective therapies including human islet transplantation, which requires strong immunosuppressants that impair beta cell survival and function. As such alternative immunomodulating therapies are required for successful transplantation. The discovery that pharmacological activation of the nuclear receptor LRH-1/NR5A2 can reverse hyperglycemia in mouse models of T1D by altering, and not suppressing the autoimmune attack, prompted us to investigate whether LRH-1/NR5A2 activation could improve human islet function/survival after xenotransplantation in immunocompetent mice. Human islets were transplanted under the kidney capsule of streptozotocin (STZ)-induced diabetic mice, and treatment with BL001 (LRH-1/NR5A2 agonist) or vehicle was administered one week post-transplant. Our study, encompassing 3 independent experiments with 3 different islet donors, revealed that mice treated for 8 weeks with BL001 exhibited lower blood glucose levels correlating with improved mouse survival rates as compared to vehicle-treated controls. Human C-peptide was detectable in BL001-treated mice at both 4 and 8 weeks indicating functional islet beta cells. Accordingly, in mice treated with BL001 for 8 weeks, the beta cell mass was preserved, while a significant decrease in alpha cells was observed compared to mice treated with BL001 for only 4 weeks. In contrast, vehicle-treated mice exhibited a reduction in insulin-expressing cells at 8 weeks compared to those at 4 weeks. These results suggest that BL001 significantly enhances the survival, engraftment, and functionality of human islets in a STZ-induced diabetic mouse model.

## Introduction

Type 1 diabetes (T1D) ranks among the most prevalent multifactorial endocrine and metabolic diseases, characterized primarily by persistent hyperglycemia. Nine million people globally have T1D of whom 1.5 million are under the age of 20 years old, causing 182,000 deaths per year (https://diabetesatlas.org/idfawp/resource-files/2022/12/IDF-Atlas-T1D-infographic.pdf). Alarming, the number of individuals with T1D is anticipated to reach 17 million by 2040 amounting to a loss of 32 years of healthy lifestyle (1). T1D is classified as either immune-mediated or idiopathic (of unknown aetiology) diabetes (2). The most common form of T1D in Western societies is immune-mediated, which is caused by a disruption in the balance between T-regulatory cells (Tregs) and T-effector cells (Teffs; CD4^+^ and CD8^+^ cytotoxic T-cells) that respond to islet-associated self-antigens. This breakdown in immune homeostasis or ‘tolerance’ leads to pancreatic islet beta cell destruction resulting in insulin deficiency and hyperglycemia (3). The only viable treatment for individuals with T1D is insulin supplementation which, despite its beneficial effects on glucose homeostasis, does not eliminate severe diabetic complications (4, 5).

Reconstitution of the beta cell mass has been a focus of intense research for the past 30 years exploring various cell types as a source for beta cell regeneration/replacement (6). As a preferred option, human islet transplantation has evolved from an experimental procedure to a standard of care for a specific group of patients, demonstrating good long-term safety and a median graft survival of 5.9 years (7). However, the shortage of donors has hindered the widespread use of this approach. Great strives have been achieved using stem cells and cellular reprograming strategies that have resulted in clinical breakthroughs (8). Combined with the recent success in the bioreactor-scalable production of stem cell-derived islets, this source offers a viable clinical implementation of islet cell therapy (9). Nonetheless, allosteric transplantation of primary and stem cell derived islets requires an aggressive immunosuppressant regimen to prevent rejection that entails secondary immune complications. In addition several post-transplant events, such as instant blood mediated inflammatory reaction and cytokine cascade, seriously affect the functionality of transplanted islets (10). Therefore alternative immunomodulatory regimen are urgently required to prevent such secondary effects permitting long-term islet integration, survival and function.

We previously demonstrated that the pharmacological activation of the nuclear receptor LRH-1/NR5A2 using a small chemical agonist (BL001) could therapeutically impede the progression of hyperglycemia in 2 mouse models of T1D (NOD and RIP-B7.1) without long-term adverse effects, validating the benefits of targeting this nuclear receptor (11). BL001-mediated activation of LRH-1/NR5A2 facilitated the resolution of the autoimmune attack *in vivo* by increasing the number of anti-inflammatory M2 macrophages and decreasing the number of pro-inflammatory M1 macrophages. Simultaneously, BL001 treatment increased the number of tolerogenic dendritic cells (tolDCs) and Tregs. Additionally, BL001 promoted beta cell regeneration through trans-differentiation and enhanced cell survival via the PTGS2/PGE2/PTGER1 signaling cascade (11–13). Given this strong tolerization capacity of LRH-1/NR5A2 activation in mouse models of T1D, and aiming for its clinical applicability, herein we sought to determine whether LRH-1/NR5A2 activation could facilitate long-term human islet engraftment and function in immunocompetent hyperglycemic mice.

## Materials and methods

### Human islet procuration

Human islets were obtained either from the Alberta Diabetes Institute IsletCore Laboratory (CA) or from ECIT-San Raffaele Scientific Institute, Milan (IT). Human islet preparations were washed, handpicked and subsequently maintained in CMRL-1066 media (ThermoFisher Scientific, Madrid, ES) containing 5.6 mM glucose, and supplemented with 10% FCS, 100 Units/ml penicillin, 100 μg/ml streptomycin and 100 μg/ml gentamycin (all purchased from Sigma-Aldrich, Madrid, ES).

### Xenotransplantation

Mouse experimentations were approved by the Andalusian Ministry of Agriculture, Farming, Fish and Sustainable Development (08/07/2019/120). Animal studies were performed in compliance with the ARRIVE guidelines (14). Eight-week-old immune-competent C57BL/6J male mice (Janvier Labs, France) were treated with a single dose of 175 mg/kg b.w. streptozotocin (STZ, Sigma-Aldrich/Merck, Spain) prepared in 0.01 M sodium citrate at pH 4.5 to induce hyperglycemia. One week later, mice were anesthetized via an intraperitoneal (*i.p.*) injection of 100 mg/Kg ketamine and 10 mg/Kg xylazine (obtained from the animal facility veterinarian) and 750 human islet equivalents (IEQ) were transplanted under the kidney capsule using a PE50 tubing connected to a 25 µL gauged Hamilton syringe. STZ-treated and transplanted mice were randomly allocated to either the vehicle- or BL001-treated group. Upon termination of the experiment, animals were euthanized and transplanted kidneys extracted, fixed and embedded for further histological analysis. The entire kidney for each independent transplantation experiment was sectioned and insulin/glucagon co-immunostaining as well as CD4 was performed at every 15^th^ slice, corresponding with an interval of ~ 75-150 µm which matches the median size of the majority of islets. The sample size for these *in vivo* studies to reach statistical significance was not precalculated because the survival of islet grafts was previously unknown. Although the xenotransplantation of islets and BL001 treatment were not blinded to the investigator, the subsequent analysis of blood samples and grafts were performed by blinded investigators.

### BL001 treatment

BL001 [(3a*S*,6a*R*)-1,2,3,3a,6,6a-hexahydro-4-(3-methoxyphenyl)-5-((*E*)-oct4-en-4-yl)-N-phenylpentalen-3a-amine] was synthesized by Sreeni Labs Private Limited (Telangana, India), at a HPLC purity > 98%. The semi-solid compound was dissolved in 100% DMSO, at 100 μg/ml stock concentrations. The optimal formulation for *in vivo* administration (hereafter referred to as vehicle) is: 1% DMSO, 40% WellSolve (Celeste Corporation, Japan) and 59% water. Mice were injected daily *i.p.* with 10 mg/kg b.w. BL001 (half-life of 9.4 hours), starting 7 days post STZ treatment.

### Glucose monitoring and health status

Circulating glucose levels were measured from tail vein blood samples using an Optium Xceed glucometer (Abbott Scientifica SA, Barcelona, Spain). An oral glucose tolerance test (OGTT) was performed at 5-weeks post-transplantation as previously described (15). The health status of mice was monitored by assessing fur appearance, wounds and tracking weight loss. Mice were euthanized if they lost more than 25% of their body weight (criteria set by the bioethics committee). Kaplan-Meier survival curve were generated based on mice that were either euthanized or found dead in the cage.

### Immunofluorescence analyses

For immunostaining, kidneys were fixed overnight in 4% paraformaldehyde at 4°C. Tissues were dehydrated, paraffin embedded, and sectioned at 5 μm thickness. Immunostaining was then performed overnight at 4°C using a combination of primary antibodies: 1) insulin (Sigma-Aldrich, Madrid, ES), 2) glucagon (Cell Signaling, Barcelona, ES) and 3) CD4 (Miltenyi Biotec, Madrid, ES) in PBS 1% BSA 0.2% TritonX100. Subsequently, secondary antibodies were incubated for 1 hour at room temperature in PBS 0.2% TritonX100. Nuclei were stained with 0.0001% of 4′,6-diamidino-2-phenylindole (DAPI, Sigma-Aldrich, Madrid, ES) and cover slips were mounted using fluorescent mounting medium (DAKO). Epifluorescence microscopy images were acquired with a Leica Thunder Imager microscope. Images of kidney sections were processed using the Thunder imager software and analyzed using the Photoshop and ImageJ (FIJI) softwares.

### Statistical analysis

Data are presented as the mean ± SEM. Student’s t-tests were used as described in the figure legend. *p* values less than or equal to 0.05 were considered statistically significant. Statistical analyses were performed using the GraphPad Prism software version 8 (GraphPad Software, La Jolla, USA).

## Results

**Figure 1:**
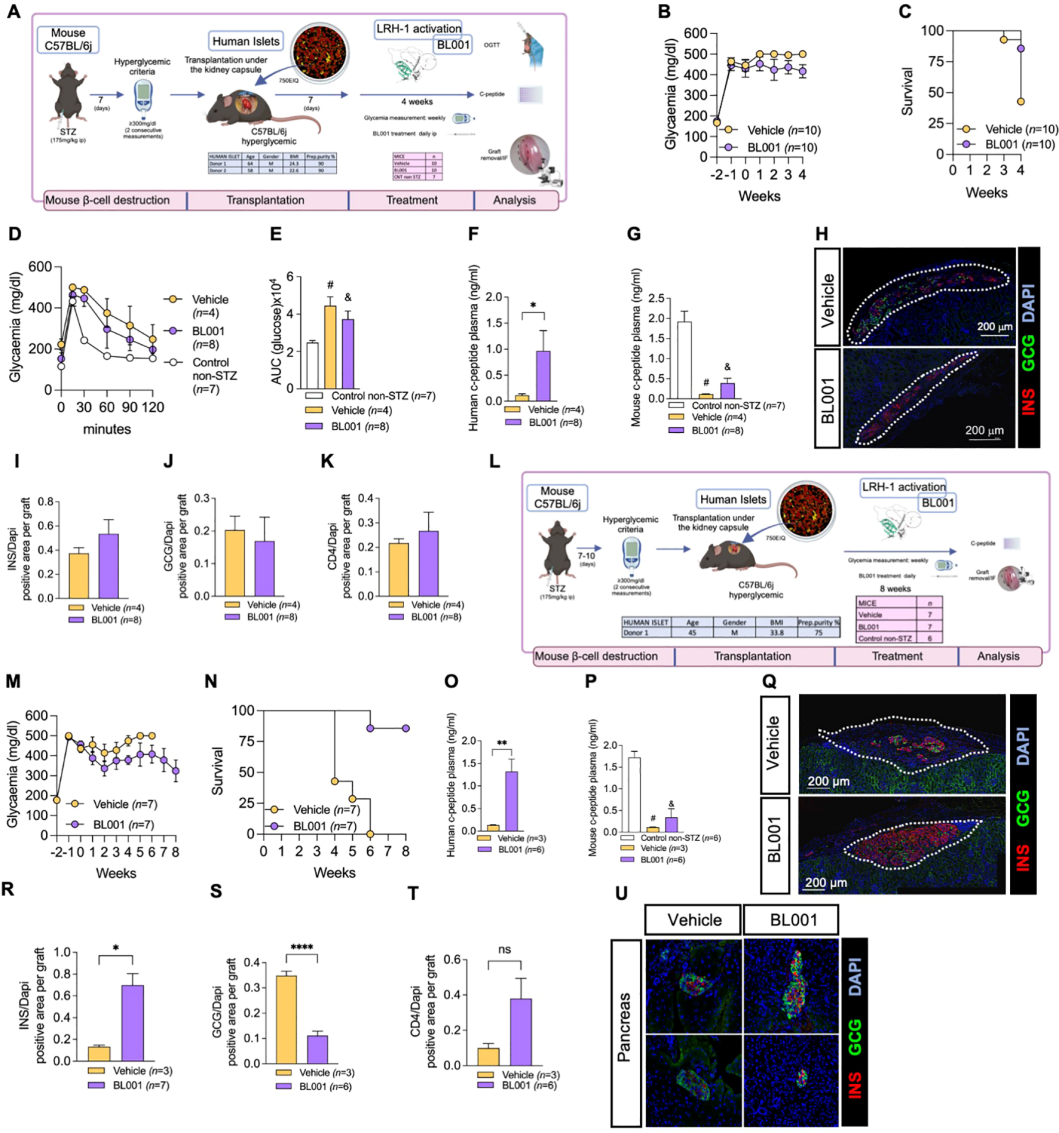
BL001 improves human islet graft survival and function in STZ-treated immunocompetent C57BL/6j mice. **(A)** Experimental design of the 4-week BL001 treatment post-xenotransplantation experiment. **(B)** Weekly measurement of non-fasting blood glucose and **(C)** Kaplan Meier survival curve. **(D)** OGTT performed at 4 weeks post-BL001/Vehicle treatment. Mice were fasted for 6 hours before the OGTT. **(E)** Area under the curve (AUC) corresponding to the OGTT. Student t-test, ^#^ *p=0003* and ^&^ *p=0.0037* as compared to control non-STZ mice. **(F)** Human C-peptide plasma levels at 4 weeks post-BL001/Vehicle treatment. Data are presented as means ± SEM. *p<0.05 student t-test. **(G)** Mouse C-peptide plasma levels at 4 weeks post-BL001/Vehicle treatment. Data are presented as means ± SEM. Student t-test, *^#^ p=002* and ^&^ *p=0.0003* as ompared to control non-STZ mice. **(H)** Representative immunofluorescence images of kidney sections from mice euthanized at 4 weeks post-BL001/Vehicle treatment, displaying staining for insulin (INS), glucagon (GCG) staining along with nuclear DAPI staining. Quantitative analysis of **(I)** insulin (INS), **(J)** glucagon (GCG), and **(K)** CD4^+^ areas, normalized to the DAPI-positive area per graft, at 4 weeks post BL001 treatment. **(L)** Experimental design of the 8-week BL001 treatment post-xenotransplantation experiment. **(M)** Weekly measurement of non-fasting glycemia and **(N)** Kaplan Meier survival curve **(O)**. Human C-peptide plasma levels at 6-week vehicle-treated mice and 8-week BL001-treated mice. Data are presented as means ± SEM. **p<0.01 student t-test. **(P)** Mouse C-peptide plasma levels at 6-week vehicle-treated mice and 8-week BL001-treated mice. Data are presented as means ± SEM. *^#^ p=0001* and ^&^ *p=0.0002* Student t-test as compared to control non-STZ mice. **(Q)** Representative immunofluorescence images of kidney sections from mice at 6 weeks post-vehicle treatment and at 8 weeks post-BL001 treatment, displaying staining for insulin (INS), glucagon (GCG) staining along with nuclear DAPI staining. Quantitative analysis of **(R)** insulin (INS), **(S)** glucagon (GCG), and **(T)** CD4^+^ areas, normalized to the DAPI-positive area per graft, at 4 weeks post BL001 treatment. Data are presented as means ± SEM. *p<0.05 and ****p<0.0001 student t-test. ns, non-significant. **(U)** Representative immunofluorescence images of pancreas sections from mice at 6 weeks post-vehicle treatment and at 8 weeks post-BL001 treatment. displaying staining for insulin (INS), glucagon (GCG) staining along with nuclear DAPI staining.

We have previously demonstrated that the pharmacological activation of LRH-1/NR5A2 using BL001 promoted immune tolerance and enhance islet cell survival in the RIP-B7.1 and NOD mouse models of T1D (11). Building on these benefits, herein we aimed to evaluate whether BL001 could improve the survival and function of human islets transplanted into hyperglycemic C57BL/6J mice. To this end, we performed suboptimal human islet transplantations (750 human islet equivalents; IEQ) under the kidney capsule of immune competent mice rendered diabetic with a single high dose of streptozotocin (STZ). One week after transplantation, mice were treated or not with BL001. The rationale for using suboptimal amounts of IEQ transplanted in immunocompetent mice was to assess whether potential improvements in human islet cell survival and insulin secretion, facilitated by the BL001 treatment, could lead to improved glycemia correlating with immune tolerance as previously reported (11). Three independent experiments were performed using islets from 3 different donors. In 2 of the experiments BL001 treatment was maintained for 4 weeks (**Figure 1A**), and in the third experiment for 8 weeks (**Figure 1L**). Although hyperglycemia persisted at either 4 and 8 weeks of treatment, blood glucose levels were lower in transplanted mice treated with BL001 for up to 8 weeks correlating with a higher survival rate as compared to vehicle treated mice at either 4 or 6 weeks, time at which mice had to be euthanized due to pre-defined health criteria (**Figures 1B, C, M, N**; **Supplementary Figure S1**). The 4-week BL001 treatment did not improve glucose tolerance in transplanted mice as compared to vehicle mice (**Figures 1D, E**). Human C-peptide was detected in BL001-treated mice, but not in vehicle-treated mice, at 4- and 8 weeks post-treatment (6 weeks for vehicle-treated), confirming functionality of the transplanted human islet (**Figures 1F, O**). In contrast, mouse C-peptide blood levels were marginally discernable in either BL001- or vehicle-STZ-treated mice, as compared to untreated mice, 4 and 6-8 weeks post-treatment (**Figures 1G, P**). Both BL001- and vehicle-treated mice sacrificed at 4-weeks retained human islet xenotransplants with a similar number of insulin and glucagon expressing cells as well as CD4^+^ T-cell infiltration (**Figures 1H–K**). The 8-week extended BL001-treatment preserved the beta cell mass in xenotransplants correlating with improved glycemia while vehicle-treated mice that were sacrificed at 6 weeks (due to pre-defined health criteria) exhibited a significantly lower number of insulin expressing cells as compared to BL001-treated mice (**Figures 1Q, R**). Interestingly, the alpha cell mass was significantly reduced in mice treated with BL001 for 8 weeks compared to those treated with the vehicle (**Figures 1Q–S**). Although not significant, CD4^+^ cytotoxic T-cell infiltration was higher in BL001-treated mice compared to vehicle-treated mice which exhibited low levels of infiltration likely due to the almost complete destruction of beta cells (**Figures 1Q–T**). Both vehicle and BL001-treated mice exhibited similar pancreatic islet insulin staining patterns, correlating with only marginally discernable mouse C-peptide blood levels in either BL001- or vehicle-STZ-treated mice, indicating that the mild recovery of glycemia in BL001-treated mice was not due to remnant endogenous islet beta cells but rather attributed to preserved islet graft mass and improved islet transplant outcomes conveyed by BL001 administration (**Figure 1U**). Taken together, these results indicate that BL001 favors the survival, engraftment, and function of a marginal mass of human islets in STZ-treated immunocompetent C57BL/6J mice.

## Discussion

Consistent with its previously reported anti-inflammatory and anti-apoptotic properties in mice (11, 12), the therapeutic administration of BL001 to hyperglycemic immunocompetent mice xenotransplanted with human islets promoted graft implantation and function, as indicated by sustained human islet beta cell mass and circulating human C-peptide levels at 4 and 8 weeks correlating with reduced hyperglycemia at 8-week post treatment. While our prior work demonstrated that short-term BL001 treatment could protect transplanted human islets in mice against rejection, long-term engraftment, and function, as evaluated by circulating human C-peptide was not assessed in that study (11). Interestingly, the alpha cell mass was increased between 4 and 8 weeks in vehicle-treated mice while it remained relatively constant in BL001-treated mice. Such increase in alpha cell mass has previously been observed in the pancreases of individuals with T1D, likely resulting from disruptions in the regulatory balance between insulin and glucagon secretion, along with changes in islet architecture and cell signaling (16, 17).

To the best of our knowledge, our study is one of the first to demonstrate that a pharmacological compound can promote human islet engraftment and function in immunocompetent mice, evading immune rejection likely via tolerization as we previously demonstrated in 2 independent mouse models of T1D (11). Most human islet transplantation survival and functional studies have been carried out in immunodeficient mice in order to circumvent rejection thereby assessing the impact of the treatment directly on transplanted cells (18). Consequently, our findings hold significant implications for human studies, as reversing the disease will not only require improved islet performance but also the resolution of the chronic pro-inflammatory/autoimmune attack predominantly steered by islet self-antigen activated macrophages and mDCs, which play a central role in the induction of T-cell expansion and subsequent beta cell destruction (3). In this context, current strategies to avoid graft rejection encompass cell encapsulation and/or aggressive immunosuppressant regimens, often resulting in secondary complications such as kidney failure (19–21). Furthermore, tacrolimus and sirolimus, both of which are components of standard immunosuppression protocols, possess diabetogenic properties (10). Other experimental strategies being explored include the combined transplantation of mesenchymal stem cells (MSCs) or Tregs with islets (22, 23). In favor of this approach we recently demonstrated that transplantation of umbilical cord MSCs delayed the onset of hyperglycemia in RIP-B7.1 mice (24). Nonetheless, this combined MSC/Tregs islet therapy merely extends graft function temporarily by delaying immune rejection, as the host eventually eliminates these cells. Alternatively, hIPSC-derived islet-like organoids are being genetically modified to successfully evade the autoimmune attack in pre-clinical mouse models (25, 26). Although promising, these approaches have yet to reach ethical approval for clinical trials. The pharmacological activation of LRH-1/NR5A2 circumvent the caveats encountered by cell therapy by attenuating the pro-inflammatory environment- without suppressing the general immune system- while increasing beta cell survival and performance (12, 13).

We acknowledge that the murine and human immune systems may respond differently to allogeneic or xenogeneic islet transplants, and that a humanized mouse model would more accurately replicate the human context. Nevertheless, we argue that both the murine and human response, regardless of the specific immune cells involved, lead to islet graft rejection, an outcome reversed by BL001-mediated activation of LRH-1/NR5A2. Furthermore, we recently demonstrated that beta cell death was reduced when co-cultured with BL001-treated human peripheral blood mononuclear cells (PBMCs) isolated from individuals with T1D, correlating with decreased cytotoxic T-cell proliferation and interferon gamma secretion (manuscript under review). In conclusion, our findings indicate the potential for a successful immunomodulatory therapy through the pharmacological activation of LRH-1/NR5A2 in humans.

## Data Availability

The raw data supporting the conclusions of this article will be made available by the authors, without undue reservation.
