# Supplementary material for: Enhancing human islet xenotransplant survival and function in diabetic immunocompetent mice through LRH-1/NR5A2 pharmacological activation
Source: Front Immunol. 2024 Sep 27;15:1470881. doi: 10.3389/fimmu.2024.1470881 (PMC11466778; doi:10.3389/fimmu.2024.1470881)
Supplement: Supplementary file 1 [file DataSheet1.pdf]

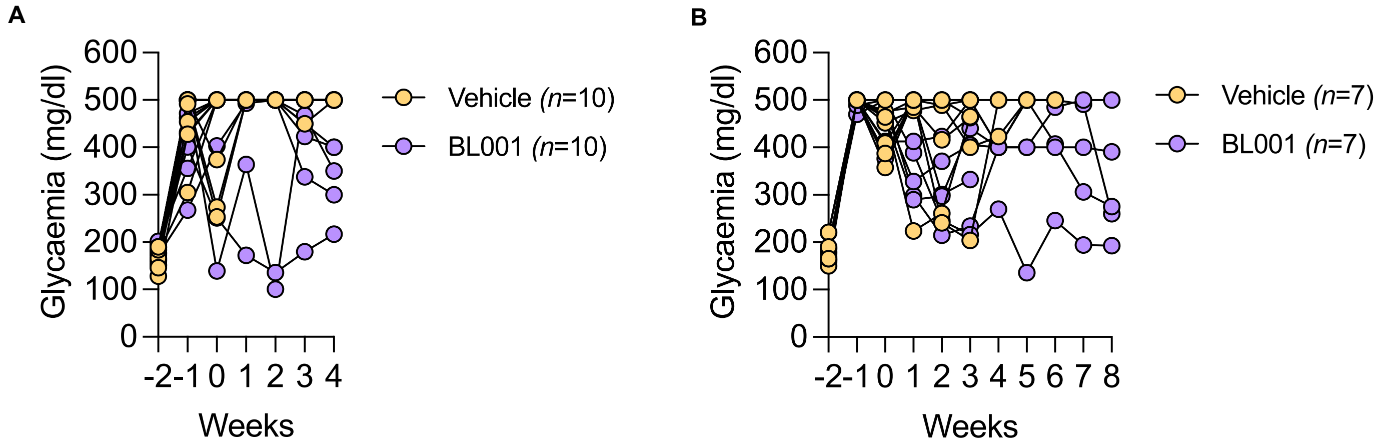

Supplementary Figure 1. Longitudinal analysis of individual blood glucose levels in mice treated with BL001 or vehicle. (A) Longitudinal measurement of non-fasting blood glucose levels for individual mice in the Vehicle-treated (n=10, orange dots) and BL001-treated (n=10; purple dots) groups, measured from 2 weeks before treatment initiation (week -2) through 4 weeks of treatment (week 4). These data correspond to the average glycemia levels presented in Figure 1B. Each line represents the glycemic profile of a single mouse. **(B)** Longitudinal measurement of non-fasting blood glucose levels for individual mice in the Vehicle-treated (n=7, orange dots) and BL001-treated (n=7, purple dots) groups, measured from 2 weeks before treatment initiation (week -2) through 8 weeks of treatment (week 8). These data correspond to the average glycemia levels presented in Figure 1M. Each line represents the glycemic profile of a single mouse.
